# Correlations Between Cognitive Functions and Clinical Symptoms in Adolescents With Complex Post-traumatic Stress Disorder

**DOI:** 10.3389/fpubh.2021.586389

**Published:** 2021-04-28

**Authors:** Yee Jin Shin, Sun Mi Kim, Ji Sun Hong, Doug Hyun Han

**Affiliations:** ^1^Department of Psychiatry, Yeonsei University Hospital, Seoul, South Korea; ^2^Department of Psychiatry, Chung-Ang University Hospital, Seoul, South Korea

**Keywords:** complex post-traumatic stress disorder, adolescents, emotional perception, spatiotemporal attention, working memory

## Abstract

**Introduction:** Complex post-traumatic stress disorder (C-PTSD) is characterized by the typical symptoms of PTSD, in addition to affective dysregulation, negative self-concept, and disturbances in interpersonal relationships. Children and adolescents with C-PTSD have been reported to have deficits in emotional and cognitive functions. We hypothesized that the following are associated with the severity of C-PTSD symptoms: (1) adolescents with C-PTSD who show deficits in emotional perception and cognitive functions, including executive function and attention; and (2) deficits in neurocognitive functions.

**Methods:** Information on 69 adolescents with PTSD, aged 10–19 years, was gathered from seven shelters. All participants were assessed using complete clinical scales, including the C-PTSD Interview and Depression, Anxiety, and Stress Scales, and neurocognitive function tests, including the emotional perception, mental rotation, and modified Tower of London tests.

**Results:** Adolescents with C-PTSD were more likely to have a history of sexual assault, dissociation, and self-harm than those with PTSD. The total and subscale scores of the C-PTSD Interview Scale in adolescents with C-PTSD were higher than that in adolescents with PTSD. In addition, neurocognitive functions, including emotional perception, attention, and working memory, were correlated with the severity of C-PTSD symptoms.

**Discussion:** Adolescents with C-PTSD experienced more serious clinical symptoms and showed more deficits in neurocognitive functions than adolescents with PTSD. Clinicians should pay careful attention toward the emotional and neurocognitive functions when assessing and treating patients with C-PTSD.

## Introduction

Complex post-traumatic stress disorder (C-PTSD) was first detailed in 2018, in the International Classification of Diseases, 11th edition. Herman initially reported C-PTSD as a condition with specific deficits in interpersonal relationships, somatization, affective regulation, dissociation, and sense of self (1). The International Statistical Classification of Diseases and Related Health Problems states that C-PTSD consists of PTSD symptoms as well as affective dysregulation, negative self-concept, and disturbances in interpersonal relationships (2).

Developmental trauma can be defined as a type of stressful event that occurs repeatedly and cumulatively over a period of time within specific relationships and contexts (3). Childhood abuse and neglect, including those with sexual, emotional, and physical components, may constitute stressful events (4). These traumatic and stressful events could be associated with several psychiatric diseases, including PTSD (5), borderline personality disorder (6), somatization disorder (7), dissociation disorders (8), self-mutilation (9), and eating disorders (10). Among the different trauma-related psychiatric diseases, C-PTSD is strongly associated with chronic and accumulated trauma from childhood (2, 11), and shows greater functional impairment (12) and poorer prognosis than PTSD (13). C-PTSD is associated with multiple types of childhood developmental and interpersonal trauma (3, 11, 12).

Several large studies support the differentiation of PTSD from C-PTSD (2, 12, 14, 15). Cloitre et al. (12) classified the treatment-seeking United State (US) population with interpersonal and single-incidence traumas into three groups: PTSD, C-PTSD, and low-symptom. In 2014, Cloitre et al. (2) also classified American female participants with a history of childhood abuse into four groups: low-symptoms, PTSD, C-PTSD, and participants with borderline personality. Elklit et al. (14), in a Danish study, classified participants (bereaved parents, rape victims, and victims of physical assault) into three groups: PTSD, C-PTSD, and low-symptoms. Perkonigg et al. (15) classified adolescent and young adult community patients in Germany into four groups: PTSD, C-PTSD, disturbance in self-organization and low PTSD, and low-symptoms.

Several studies have suggested that specific symptoms, such as affective dysregulation, negative self-concept, and disturbance in interpersonal relationships, may be associated with emotional and cognitive dysfunction in patients with C-PTSD (16–19). Adolescents with C-PTSD have a higher prevalence of depressive and anxiety disorders, such as panic disorder and general anxiety disorder (19). Relative to a depressive mood, anxiety is common in both PTSD and C-PTSD (18). In 1346 adults of East Asian origin, aged 18–24 years, Ho et al. (17) reported that anxiety was associated with both C-PTSD and PTSD, but affective dysregulation was significantly associated only with C-PTSD. By merging the findings of the aforementioned studies, it can be concluded that affective dysregulation could be a symptom that differentiates C-PTSD from PTSD.

Individuals with negative personal experiences report worse psychological well-being and difficulty in autobiographical memory coherence (20). In this context, working memory capacity could affect the concept of self by mediating the association of mood with memory coherence (21). In a systematic review evaluating the cognitive functions of children after natural disasters and/or terrorist attacks, Pfefferbaum et al. (22) reported that experiencing such events in childhood could negatively affect attention, concentration, memory, academic achievement, and executive function. Furthermore, Rivera-Velez et al. (23) reported memory and executive function deficits in 12 women with a history of childhood sexual abuse. Social skill training has been shown to improve social interactions of children with attention problems (24). In this aspect, assessment of working memory and attention could predict the progress and status of patients with C-PTSD.

Based on the clinical characteristics of dysfunction in affective regulation, negative self-concept, and difficulty with interpersonal relationships in patients with C-PTSD, we hypothesized the following: (1) adolescents with C-PTSD have deficits in emotional perception and cognitive functions, including executive function and attention; and (2) deficits in neurocognitive functions are associated with the severity of C-PTSD symptoms.

## Methods

### Participants

From 2017 to 2019, the Korean Association against Violence and Abuse, a corporation under the Secretariat of the National Assembly, sent letters to 10 state shelters that host adolescents with a history of parental abuse to offer art therapy for adolescents with C-PTSD. The criteria for participation in the program were as follows: (1) 13–24 years of age, (2) voluntary participation, and (3) PTSD diagnosis (non-complex or complex). Seven shelters accepted to participate. Among the 127 adolescents at these seven shelters, 102 (80.3%) agreed to participate in the program and 25 refused. Before providing art therapy, diagnosis and neurocognitive intervention was provided. Two psychiatrists (Y.J. Shin and J.S. Hong) interviewed all adolescents to assess and diagnose them; 73/102 (71.6%) participants were diagnosed with a form of PTSD by both psychiatrists.

Of the 73 participants with PTSD, 43 had symptoms that checked more than four of the seven C-PTSD-I domains (25); 39 were diagnosed with C-PTSD by both psychiatrists, and four by only one psychiatrist, who were subsequently excluded from the analysis. Eventually, a total of 39 adolescents with C-PTSD and 30 adolescents with PTSD were recruited for the current analyses.

The art therapy program consisted of 16 sessions, described as follows: sessions 1–2: introduction to art therapy and rapport; session 3–6: affecting recognition and regulation, setting goals, and making emotion books using line, color, and face; sessions 7–8: cognition reconstruction; sessions 9–10: interpersonal relationships; sessions 11–13: making a new self-image and wish tree using gypsum; and closing programs: drawing a person in the rain.

After four diagnostic training sessions, the diagnostic concordance rate between the two doctors was 0.80. To differentiate co-occurring psychiatric diseases, the Korean version of the Mini International Neuropsychiatric Interview was used (26). In addition, a psychologist estimated the cognitive and emotional status of all adolescents. We extracted the psychological and emotional assessment results from these data. The ethics committee of Yeonsei University approved this study (IRB number: 4-2019-0156). Written informed consent was provided by participants aged 16 years or older or by the legal guardians of participants aged below 16 years.

### Measures

#### Clinical Scales

##### C-PTSD Interview Scale

The symptoms of C-PTSD were assessed using the Korean version of the C-PTSD Interview Scale (C-PTSD-I) (25, 27). It consists of 37 items, including the following seven domains: regulation of affect and impulse, alterations in attention or consciousness, alteration in self-conception, alteration in perception of the perpetrator, alteration in relationships with others, somatization, and alteration in meaning system. The internal consistency of the C-PTSD-I Korean version was 0.971 (Cronbach's alpha).

##### Depression, Anxiety, and Stress Scale

The Depression, Anxiety, and Stress Scale (DASS) was developed to measure changes in the emotional state of patients with PTSD (28, 29). The original version of the DASS consists of 42 items, but a short version—the DASS-21—was developed for screening PTSD symptoms at different levels of depression, anxiety, and stress (28, 29). Higher DASS-21 scores reflect higher levels of depression, anxiety, and stress. The correlation between the DASS-21 scores, Beck Depression Inventory, and Beck Anxiety Inventory was 0.79–0.85.

#### Neurocognitive Function Tests

Neurocognitive functions in all adolescents were estimated using the modified Tower of London test, the emotional perception test, and the mental rotation test (CNT-MBI®, Seoul, South Korea). These tests have been used for assessing cognitive and emotional perceptions in professional gamers and pro-baseball players (30).

**Figure 1:**
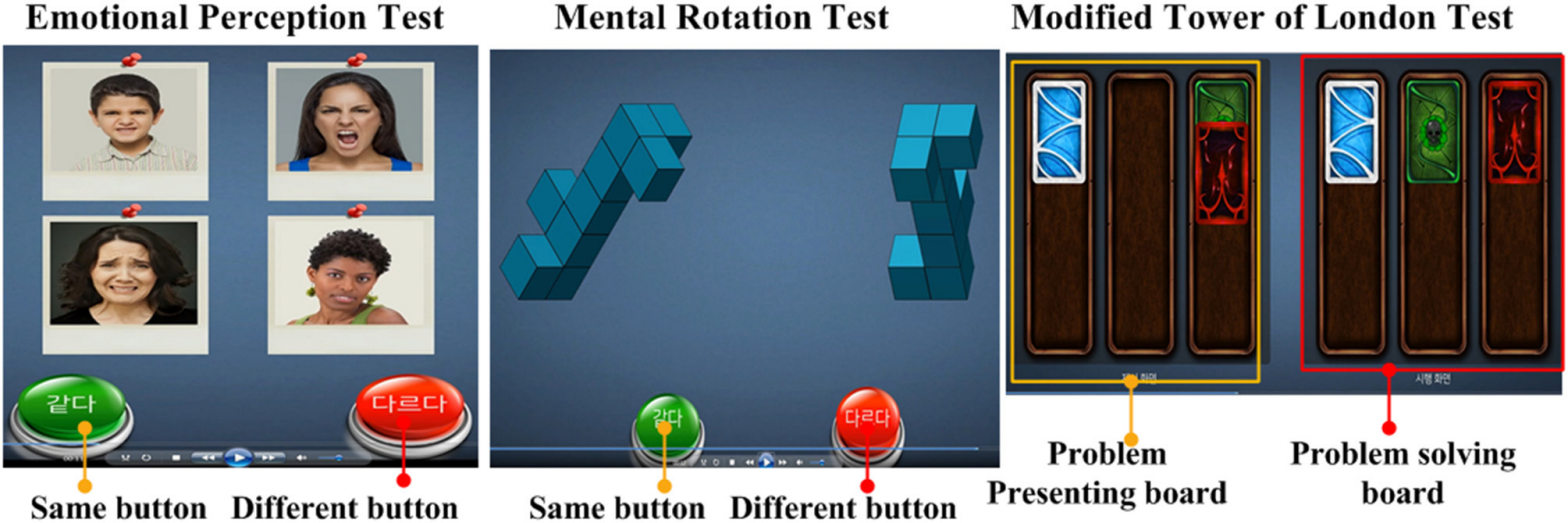
Neurocognitive function test. The images were obtained from the neurocognitive function test manufacturer MBI® (https://www.mbi-clinic.center/).

The emotional perception test consists of 108 questions regarding whether the same or different faces are presented. In each question, 2–8 faces (pleasant, unpleasant, and neutral faces) are presented on the screen in each trial. The subject then pushes the “same” or “different” button in response to the presented faces. During the test, the mean accuracy rate and reaction time from the presentation of the pictures to the pushing of the buttons were recorded. It has a test–retest reliability of 0.81 (Figure 1). Faster reaction times and more correct responses in the emotional perception test represent better emotional perception (31).

In the mental rotation test, a pair of three-dimensional (3D) objects is shown on a screen; these objects are rotated along a certain axis by 0, 60, 90, 120, or 180°. In some presentations, the shape of the two objects are the same but rotated, while in some trials, the shapes are different. The subjects are asked to judge whether the two 3D objects are the same. During the test, the mean accuracy rate and reaction time from the presentation of the pictures to a response was recorded. It has a test–retest reliability of 0.79 (Figure 1). Faster reaction times and more correct responses in the mental rotation test represent better spatiotemporal visual attention (32).

In the modified Tower of London test, there are two boards with three lines and cards of different colors. Classically, the Tower of London test was invented to assess working memory for planning (33). The modified Tower of London test shows two boards (problem-presenting board and problem-solving board) on a screen with three lines and several cards of different colors. Using the cards on the boards, the computer presents a problem-solving task to the subject. The subject moves the cards on the problem-solving board to match the order of the cards shown on the problem-presenting board. During the test, the number of cards moved and the time taken to solve the problem were recorded. It has a test–retest reliability of 0.82 (Figure 1). Faster reaction times and a lower number of moves are associated with better working memory (33, 34).

### Statistical Analysis

Normality of the data distribution was confirmed with the Shapiro–Wilk-test. Differences in demographic characteristics, history of trauma, and comorbid diseases between the C-PTSD and PTSD groups were analyzed using the independent samples *t*-test and chi-square-test. Statistical significance was set at *p* < 0.05. Differences between the two groups in the C-PTSD-I and DASS scores were analyzed using an independent samples *t*-test. The statistical significance for the total C-PTSD-I score was set at *p* < 0.05, but significance for subscale scores of C-PTSD-I was set at *p* < 0.007 (0.05/7). The statistical significance of the DASS score was set at *p* < 0.017 (0.05/3). Differences in the results of neurocognitive function tests, including the emotional perception test, modified Tower of London test, and mental rotation test, were analyzed using independent samples *t*-test. To assess the effects of biological sex on C-PTSD, differences in C-PTSD-I scores, DASS scores, and neurocognitive function tests, an analysis was performed using the analysis of covariance and regression (ANCOVA) test, controlling for biological sex. The statistical significance for the neurocognitive function tests was set at *p* < 0.017 (0.05/3).

Correlations between C-PTSD-I scores and neurocognitive function test results were assessed using partial correlations, controlling for age and years of education. Statistical significance was set at *p* < 0.017 (0.05/3).

## Results

### Normality of Data

All data, including age, education years, C-PTSD-I Korean subscale scores (including affect and impulse, attention or consciousness, self-perception, perception of perpetrator, relations with others, somatization, and meaning), DASS subscale scores (including depression, anxiety and stress, emotional perception test–reaction time, emotional perception test–accuracy rate, mental rotation–reaction time, and mental rotation–accuracy rate), Tower of London test–reaction time, and Tower of London test–card numbers were slightly skewed and kurtotic for both C-PTSD and PTSD groups. All data was found to be normally distributed for analyses.

### Comparison of Demographic Characteristics and Clinical Scales Between the Complex and Non-complex Post-traumatic Stress Disorder Groups

Among the 69 adolescents with a history of childhood abuse, 39 (56.5%) were diagnosed with C-PTSD. Compared to the PTSD group, the C-PTSD group showed higher rates of a history of sexual assault (36.7 vs. 71.8%), dissociation (6.7 vs. 28.2%), and self-harm (3.3 vs. 30.8%) (Table 1). The duration of sexual assault in the C-PTSD group was longer than that in the PTSD group (6.64 ± 3.75 years vs. 2.72 ± 1.27 years; Table 1). There were no significant differences in age, sex ratio, or years of education between the C-PTSD and PTSD groups.

**Table 1:** Comparison of demographic characteristics and clinical scales between the C-PTSD and PTSD groups.

	**C-PTSD group** **(*n* = 39)**	**PTSD group** **(*n* = 30)**	**Statistics**
Age (years)	16.28 ± 3.11	14.86 ± 3.43	*t* = 1.73, *p* = 0.09
Sex (male/female)	9/30	13/17	χ^2^ = 3.20, *p* = 0.12
Years of education	9.45 ± 2.20	8.21 ± 2.93	*t* = 1.80, *p* = 0.08
Physical assault history	39 (100%)	30 (100%)	
Neglect history	29 (74.4%)	17 (56.7%)	χ^2^ = 2.39, *p* = 0.13
Sexual assault history	28 (71.8%)	11 (36.7%)	χ^2^ = 8.52, *p* < 0.01^*^
Assault duration (years)	6.64 ± 3.75	2.72 ± 1.27	*t* = 3.36, *p* < 0.01^*^
**Comorbid disorders**			
Dissociation	11 (28.2%)	2 (6.7%)	χ^2^ = 5.14, *p* = 0.03^*^
Mood disorder	21 (53.8%)	14 (46.7%)	χ^2^ = 0.35, *p* = 0.63
Low intelligence	20 (51.3%)	14 (46.7%)	χ^2^ = 0.15, *p* = 0.81
Conduct disorder	2 (5.1%)	2 (6.7%)	χ^2^ = 0.07, *p* < 1.00
Anxiety disorder	15 (38.5%)	11 (36.7%)	χ^2^ = 0.02, *p* < 1.00
Psychotic disease	0	0	
Eating disorder	3 (7.7%)	1 (3.3%)	χ^2^ = 0.59, *p* = 0.63
Self-harm	12 (30.8%)	1 (3.3%)	χ^2^ = 8.34, *p* < 0.01^*^

* *Statistical significance*.

*C-PTSD, complex post-traumatic stress disorder*.

Adolescents in the C-PTSD group had higher total scores (35.82 ± 26.90 vs. 18.50 ± 17.42) and exhibited significant differences in affect and impulse (9.43 ± 7.07 vs. 4.64 ± 4.57), attention or consciousness (7.43 ± 5.81 vs. 3.33 ± 2.85), self-perception (9.28 ± 7.27 vs. 3.63 ± 5.95), relationships with others (6.51 ± 5.81 vs. 2.83 ± 2.71), and meaning system (2.10 ± 2.08 vs. 0.63 ± 1.21) on the C-PTSD-I than those in the PTSD group (Table 2). Adolescents in the C-PTSD group had higher DASS scores for depression (7.86 ± 6.07 vs. 4.08 ± 4.75) and stress (8.41 ± 5.57 vs. 4.96 ± 4.86) than those in the PTSD group (Table 2). In the ANCOVA test controlling for biological sex, the results of complex PTSD-I scores and DASS scores were the same as those observed in the independent *t*-test.

**Table 2:** Comparison of complex PTSD-I and DASS scores between the complex and non-complex PTSD groups.

	**C-PTSD** **(*n* = 39)**	**PTSD group** **(*n* = 30)**	**Statistics**
**C-PTSD-I Korean version**			
Total	35.82 ± 26.90	18.50 ± 17.42	*t* = 2.81, *p* = 0.007^*^
Affect and impulse	9.43 ± 7.07	4.64 ± 4.57	*t* = 3.14, *p* = 0.003^*^
Attention or consciousness	7.43 ± 5.81	3.33 ± 2.85	*t* = 3.22, *p* < 0.001^*^
Self-perception	9.28 ± 7.27	3.63 ± 5.95	*t* = 3.20, *p* = 0.001^*^
Perception of perpetrator	2.39 ± 2.50	1.05 ± 1.87	*t* = 2.20, *p* = 0.032
Relations with others	6.51 ± 5.81	2.83 ± 2.71	*t* = 2.87, *p* = 0.006^*^
Somatization	1.35 ± 2.44	1.67 ± 2.51	*t* = −0.46, *p* = 0.654
Meaning	2.10 ± 2.08	0.63 ± 1.21	*t* = 3.152, *p* = 0.003^*^
**DASS score**			
DASS depression	7.86 ± 6.07	4.08 ± 4.75	*t* = 2.59, *p* = 0.014^*^
DASS anxiety	6.51 ± 6.13	4.29 ± 4.48	*t* = 1.49, *p* = 0.141
DASS stress	8.41 ± 5.57	4.96 ± 4.86	*t* = 2.50, *p* = 0.015^*^

* *Statistical significance*.

*C-PTSD-I, Complex post-traumatic stress disorder Interview Scale; DASS, Depression, Anxiety, and Stress; PTSD, post-traumatic stress disorder*.

*Affect and impulse, alterations in regulation of affect and impulse; Attention or consciousness, alterations in attention or consciousness; Self-perception, alterations in self-perception; Perception of perpetrator, alterations in perception of perpetrator; Relations with others, alterations in relations with others; Meaning, alterations in meaning system*.

### Comparison of Neurocognitive Functions Between the Complex and Non-complex Post-traumatic Stress Disorder Groups

Adolescents in the C-PTSD group had a lower accuracy rate in the emotional perception test (0.66 ± 0.15 vs. 0.74 ± 0.11) and the mental rotation test (0.64 ± 0.15 vs. 0.74 ± 0.15) and a higher number of moved cards in the modified Tower of London test (9.16 ± 2.07 vs. 7.81 ± 2.19) than those in the PTSD group (Table 3).

**Table 3:** Comparison of neurocognitive functions between the complex and non-complex PTSD groups.

	**C-PTSD group** **(*n* = 39)**	**PTSD group** **(*n* = 30)**	**Statistics**
**EP Test**			
EP-RT (second)	3.78 ± 1.69	3.91 ± 1.02	*t* = −0.37, *p* = 0.71
EP-AR	0.66 ± 0.15	0.74 ± 0.11	*t* = −2.69, *p* = 0.009^*^
**MR Test**			
MR-RT	3.86 ± 2.40	3.70 ± 2.12	*t* = 0.27, *p* = 0.785
MR-AR	0.64 ± 0.15	0.74 ± 0.15	*t* = −2.71, *p* = 0.008^*^
**Modified ToL Test**			
ToL-RT	14.08 ± 6.1	12.1 ± 4.38	*t* = 1.49, *p* = 0.139
ToL-CN	9.16 ± 2.07	7.81 ± 2.19	*t* = 3.07, *p* = 0.003^*^

* *Statistical significance*.

*C-PTSD, complex post-traumatic stress disorder; AR, accuracy rate; CN, number of moved cards; EP, emotional perception; MR, mental rotation; RT, reaction time; ToL, Tower of London*.

In the ANCOVA test controlling for sex, the results of the neurocognitive function tests were the same as those observed in the independent *t*-test.

### Correlations Between Symptoms of Complex Post-traumatic Stress Disorder and Results of Neurocognitive Function Test

**Figure 2:**
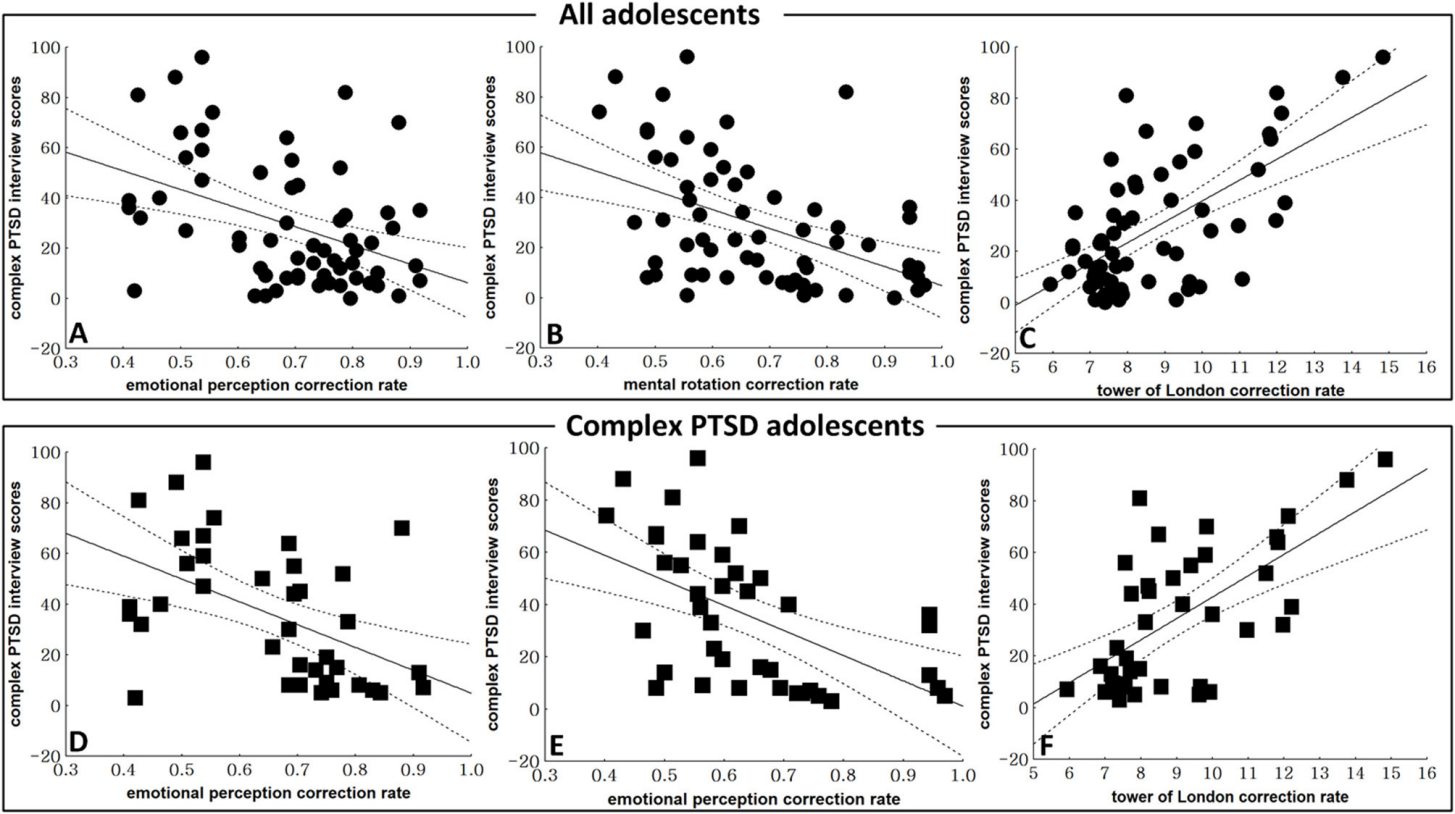
Correlations between C-PTSD symptoms and neurocognitive function test results. **(A)** The correlation between total complex post-traumatic stress disorder (C-PTSD) Interview Scale scores and the accuracy rate in the emotional test (*r* = −0.414, *p* = 0.001) in all adolescents (C-PTSD and PTSD groups). **(B)** The correlation between the total C-PTSD Interview Scale and the accuracy rate in the mental rotation test (*r* = −0.468, *p* < 0.001) in all adolescents. **(C)** The correlation between the total C-PTSD Interview Scale and the number of moved cards in the modified Tower of London test (*r* = 0.631, *p* < 0.001) in all adolescents. **(D)** The correlation between the total C-PTSD Interview Scale scores and the accuracy rate in the emotional test (*r* = −0.497, *p* = 0.001) in the C-PTSD group. **(E)** The correlation between total C-PTSD Interview Scale scores and the accuracy rate in the mental rotation test (*r* = −0.542, *p* < 0.001) in the C-PTSD group. **(F)** The correlation between the total C-PTSD Interview Scale scores and the number of moved cards in the modified Tower of London test (*r* = 0.636, *p* < 0.001) in the C-PTSD group.

The total C-PTSD-I scores of all the participants (C-PTSD and PTSD groups) correlated negatively with the accuracy rate in the emotional perception test (*r* = −0.414, *p* = 0.001) and mental rotation test (*r* = −0.468, *p* < 0.001), but correlated positively with the number of moved cards in the modified Tower of London test (*r* = 0.631, *p* < 0.001; Figure 2).

In the C-PTSD group, the total C-PTSD-I scores correlated negatively with the accuracy rate in the emotional perception test (*r* = −0.497, *p* = 0.001) and the mental rotation test (*r* = −0.542, *p* < 0.001), and positively with the number of moved cards in the modified Tower of London test (*r* = 0.636, *p* < 0.001; Figure 2). In the PTSD group, there were no significant correlations between the total C-PTSD-I scores and the neurocognitive function test results.

## Discussion

The results of this study show that adolescents with C-PTSD have a higher rate of a history of sexual assault, dissociation, and self-harm than those with PTSD. The total and subscale scores of the C-PTSD-I, including affect and impulse, attention, self-perception, perception of perpetrator, relationship with others, and meaning in adolescents with C-PTSD, were higher than those in adolescents with PTSD. In addition, neurocognitive functions, including emotional perception, attention, and working memory, correlated with the severity of C-PTSD symptoms.

### Comparison of Demographic Characteristics and Clinical Scales Between the Complex and Non-complex Post-traumatic Stress Disorder Groups

In this study, adolescents with C-PTSD had a higher rate of history of sexual assault, dissociation, and self-harm than those with PTSD. As a core symptom of C-PTSD, affective dysregulation can differentiate C-PTSD from PTSD. Among the different trauma stressors, sexual assault is thought to be the most likely cause of psychopathology and emotional dysregulation in patients with PTSD (4, 5). Hyland et al. (35) reported that patients with C-PTSD have higher levels of dissociative experiences than those with PTSD and healthy individuals. Classically, a history of childhood sexual and physical assault is thought to be a predictive factor for self-destructive behaviors in adults (9). Villalta et al. (36) reported that self-organization disturbance in adolescents with C-PTSD is strongly associated with sexual assault.

In the current study, adolescents with C-PTSD had higher total C-PTSD-I scores as well as differences in the affect and impulse, attention or consciousness, self-perception, relationship with others, and meaning system subscales than those with PTSD. In addition, adolescents in the C-PTSD group had higher DASS depression and DASS stress scores than those in the PTSD group. As the definition of C-PTSD includes affective dysregulation, negative self-concept, and disturbances in relationships (2), the functional deficits due to clinical symptoms were represented in the total and five subscale scores of C-PTSD-I in our sample. In a study on North Korean defectors with a history of trauma, Kim (37) reported the symptoms of C-PTSD using the total and seven subscale scores of the C-PTSD-I. Interestingly, there were no significant differences in the DASS anxiety scores between C-PTSD and PTSD in the current study. Prominent affective dysregulation and the similarity in anxiety scores between C-PTSD and PTSD were consistent with the findings of previous studies (17, 18).

In ANCOVA analysis controlling for biological sex, the results of C-PTSD-I scores, DASS scores, and neurocognitive functions were the same as those observed in the independent *t*-test. Other study has not reported the effects of biological sex on C-PTSD (11). However, the respective numbers of participants in those studies were small. Future studies should consider the biological sex effects on C-PTSD using a balanced number of male and female participants in a larger cohort.

### Comparison of Neurocognitive Functions Between the Complex and Non-complex Post-traumatic Stress Disorder Groups

The results of neurocognitive functional assessments showed that the emotional perception, mental rotation, and Tower of London tests in the C-PTSD group were worse than those in the PTSD group. In addition, all neurocognitive functions correlated with the severity of C-PTSD symptoms. Emotional dysregulation and deficits in emotional perception are well-known C-PTSD symptoms (16, 19). The lower accuracy scores of the emotional perception test already represented the dysfunction of emotional perception in previous studies (38). Affective dysregulation assessed using the neurocognitive function test in C-PTSD would also be more prominent than that in PTSD.

Inattention and intrusive visual memory dysfunction are well-known symptoms of PTSD (34, 35). Intrusive visual memories are core PTSD symptoms (39). Inattention induced by intrusive visual memories is a key target for early intervention in patients with a history of trauma (40). However, inattention is also thought to be a crucial factor in interpersonal relationships. Difficulty in interpersonal relationships is another key factor in C-PTSD (19). In the current study, we found more deficits in spatiotemporal visual attention, which were assessed using the mental rotation test, in adolescents with C-PTSD than in those with PTSD.

PTSD symptoms, including re-experiencing the traumatic event, avoidance, and hyperarousal in maltreated youth, are associated with a deficit in working memory (41). In the current study, we found more deficits in working memory, which was assessed using the modified Tower of London test, in adolescents with C-PTSD than in those with PTSD. Working memory is thought to mediate the association between mood and negative self-concept (21). Working memory training is considered effective for emotional regulation in the treatment of patients with PTSD (42). Affective dysregulation and negative self-concept are the main symptoms of C-PTSD. For these reasons, the deficit of working memory in C-PTSD might be more serious than that observed in PTSD.

## Limitations

This study has a few limitations. First, the number of subjects was limited to confidently generalize the study results. Moreover, we could not link the effect of sexual assault on C-PTSD symptoms because of the small number of subjects. Second, the diagnosis of participant was established by psychiatrists only using clinical ratings, without psychometric diagnostic interview. Therefore, readers should be cautious with interpreting the results. Finally, the family environment of adolescents with C-PTSD could not be investigated in the current study. Future studies should consider the family environment and a large number of participants.

## Conclusions

Because adolescents with C-PTSD showed more deficits in clinical symptoms and cognitive functions than those with PTSD, psychiatrists should pay careful attention toward neurocognitive function when assessing patients with C-PTSD. In addition, regulation of emotion and improving working memory could be crucial factors for treating C-PTSD.

## Data Availability Statement

The raw data supporting the conclusions of this article will be made available by the authors, without undue reservation.

## Ethics Statement

The studies involving human participants were reviewed and approved by The Ethics Committee of Yeonsei University. Written informed consent to participate in this study was provided by the participants' legal guardian/next of kin.

## Author Contributions

YS and DH: conceptualization. DH and SK: methodology. SK: formal analysis. JH: investigation. DH: writing—original draft preparation. YS: writing—review, editing, and supervision. All authors approved the final manuscript as submitted and agree to be accountable for all aspects of the work.

## Conflict of Interest

The authors declare that the research was conducted in the absence of any commercial or financial relationships that could be construed as a potential conflict of interest.
